# Fisetin Attenuates Diabetic Nephropathy-Induced Podocyte Injury by Inhibiting NLRP3 Inflammasome

**DOI:** 10.3389/fphar.2022.783706

**Published:** 2022-01-21

**Authors:** Wenmin Dong, Chenglin Jia, Ji Li, Yi Zhou, Yun Luo, Jibo Liu, Zhiguo Zhao, Jiaqi Zhang, Shan Lin, Ying Chen

**Affiliations:** ^1^ Shanghai TCM-Integrated Hospital Affiliated to Shanghai University of Traditional Chinese Medicine, Shanghai, China; ^2^ Shanghai University of Traditional Chinese Medicine, Shanghai, China

**Keywords:** fisetin, diabetic nephropathy, podocyte injury, inflammasome, autophagy

## Abstract

Diabetic nephropathy (DN) is one of the primary complications of diabetes. Fisetin is a flavonoid polyphenol that is present in several vegetables and fruits. The present study investigated the mechanisms of fisetin in DN-induced podocyte injury both *in vitro* and *in vivo*. The results revealed that fisetin ameliorated high glucose (HG)-induced podocyte injury and streptozotocin (STZ)-induced DN in mice. *CDKN1B* mRNA expression in the glomeruli of patients with DN decreased based on the Nephroseq dataset, and fisetin reversed *CDKN1B* expression at mRNA and protein levels in a dose-dependent manner in podocytes and mice kidney tissues. Furthermore, fisetin suppressed the phosphorylation of P70S6K, a downstream target of *CDKN1B*, activated autophagosome formation, and inhibited Nod-like receptor protein 3 (NLRP3) inflammasomes. Interfering *CDKN1B* reduced the protective effects of fisetin against high glucose-induced podocyte injury. Molecular docking results revealed a potential interaction between fisetin and *CDKN1B*. In summary, the present study revealed that fisetin alleviated high glucose-induced podocyte injury and STZ-induced DN in mice by restoring autophagy-mediated CDKN1B/P70S6K pathway and inhibiting NLRP3 inflammasome.

## Introduction

Diabetic nephropathy (DN) is one of the chronic and severe microvascular complications of diabetes, and a major cause of end-stage renal disease (ESRD). Current epidemiological statistics indicate that the global prevalence of diabetes mellitus (DM) is increasing annually, with a considerable increase from 108 million in 1980 to 451 million in 2017, and the number has continued to rise (Carracher et al., 2018). According to the International Diabetes Federation, the number of people with diabetes globally was estimated to be 463 million in 2019, and is expected to increase to 578 million by 2030 and to 700 million by 2045. Approximately 30–40% of the people with diabetes develop DN (Saeedi et al., 2019), which predicts a dramatic increase in the number of people with DN. As a progressive endpoint of DN, ESRD requires haemodialysis to maintain the lifeline of patients and places a huge burden on the society and families. Therefore, the early detection and management of DN is particularly important in the development of DN (Finkelstein et al., 2012; Sumida and Kovesdy, 2017).

Podocytes are the outermost cells of the glomerular filtration barrier. In diabetic cases, hyperglycaemia, angiotensin, and TGF-β can induce apoptosis, transdifferentiation, abnormal secretory function, increased extracellular matrix synthesis, cytoskeletal rearrangement, and other damaging changes in podocytes, thereby disrupting the integrity of the glomerular filtration barrier and eventually leading to proteinuria. Intracellular oxidative stress and inflammatory responses are pathophysiological mechanisms through which such injuries occur (Spurney and Coffman, 2008; Dong et al., 2021). Both cellular biological effects and haemodynamic alterations caused by impaired glucose metabolism in diabetic patients can cause damage to renal intrinsic cells, releasing multiple inflammatory mediators, which, in turn, promote the activation of monocyte aggregates and activate inflammatory responses. The alterations further damage renal intrinsic cells, leading to increased endothelial cell permeability, phenotypic transformation or apoptosis of podocytes and tubular epithelial cells, proliferation of thylakoid cells, and increased production of extracellular matrix, which result in more progressive pathological and structural changes in the kidney (Wada and Makino, 2013).

Recent studies have revealed that most fruits and vegetables are rich in flavonoids, which have anti-inflammatory and antioxidant effects (Gao et al., 2019; Zhang et al., 2020a; Zhao et al., 2020; Zhang et al., 2021). The hypoglycemic effects of flavonoids are associated with mechanisms, such as improved insulin resistance, inhibition of non-enzymatic glycosylation reactions of proteins, and reduction of blood lipids and oxidative stress levels (Sharma et al., 2015; Williamson, 2017). Fisetin is a natural dietary flavonoid largely found in various fruits and vegetables, such as apples, persimmons, grapes, strawberries, cucumbers, and onions. Pharmacological studies conducted over the last few years have revealed that fisetin has strong anti-inflammatory, antioxidant, anti-tumour, and protective effects against myocardial and cerebral ischaemic injury (Dong et al., 2018; Liu et al., 2019; Zhao et al., 2019; Zhang et al., 2020b). Fisetin has been widely reported to have the effect of improving several types of kidney injuries (Ge et al., 2019; Ren et al., 2020; Chenxu et al., 2021). However, the mechanisms of its actions against DN-induced podocyte injury remain unclear. In this article, we will used high glucose induced podocytes injury model. Previous studies confirmed that reduced eNOS activity enhances glomerular mesangial matrix expansion and podocyte foot process effacement in the setting of diabetes and was shown to worsen diabetic nephropathy that better resembles human diabetic nephropathy phenotype in mice (Kanetsuna et al., 2007; Zhong et al., 2019). STZ is known to have nonspecific toxic effects on a variety of tissues, including renal tissues (Breyer et al., 2005), so it is possible that eNOS deficiency may enhance toxicity of STZ to cause glomerular endothelial damage. Hence, STZ was administered in eNOS-null mice (+STZ) to build T1DM model and fisetin will be used to explore its effect on diabetic nephropathy.

## Materials and Methods

### Animals

Male eNOS homozygous knockout (eNOS−/−) mice (18–22) with C57BL/6J background were purchased from Caygen Biosciences Inc. (Guangzhou, China) and housed under specific pathogen-free conditions. The present study was carried out in strict accordance with the Guide for the Care and Use of Laboratory Animals (Eighth Edition, 2011, published by The National Academies Press, 2101 Constitution Ave. NW, Washington, DC 20055, United States). The protocol was reviewed and approved by the Animal Care Committee of Shanghai TCM-Integrated Hospital (Permit Number: PZSHUTCM201204008). Diabetes was induced in 8-week old mice through intraperitoneal (I.P.) administration of streptozotocin (STZ) (S0130, dissolved in 0.1 M citrate buffer, pH 4.5; Sigma-Aldrich, St. Louis, MO, United States) at a dose of 50 mg/kg after 4–6 h of food deprivation daily for five consecutive days. Non-diabetic controls were injected with citrate buffer. Ten weeks after induction of diabetes, mice were treated with fisetin (5, 10, or 20 mg/kg), which was purchased from Shanghai Yuanye Bio-Technology Co., Ltd. (Shanghai, China) that was orally administered every 2 days for 8 weeks. Mice that received 5% dimethyl sulfoxide (DMSO) served as controls for the lentivirus treatments. Mice were euthanized 18 weeks after the onset of diabetes and the kidneys harvested for subsequent experiments. Surgery was performed under sodium pentobarbital anesthesia, and all efforts were made to minimize suffering.

### Assessment of Urine Albumin

Urine albumin was analyzed using ELISA kits (Nanjing Jiancheng Bioengineering Institute, Nanjing, China) according to the manufacturer’s protocols.

### Cell Culture

Conditionally immortalized mouse podocytes were obtained from the Cell Bank at the Chinese Academy of Sciences (Shanghai, China) and cultured in DMEM (Dulbecco’s Modified Eagle Medium) containing 10% fetal calf serum.

### Statistical Analysis

Statistical analyses were carried out using the program R (www.r-project.org). All data were expressed as means ± standard error of the mean (SEM). Significant differences in mean values among groups were evaluated using one-way analysis of variance, followed by least significant difference post hoc tests for mean separation. Comparisons between two groups were performed using Student’s *t*-test (two tailed). Significance level was set at *p* < 0.05.

Detailed information with regard to the materials and methods is presented in the Supplementary Material.

## Results

### Fisetin Ameliorates High Glucose-Induced Podocyte Injury

**FIGURE 1:**
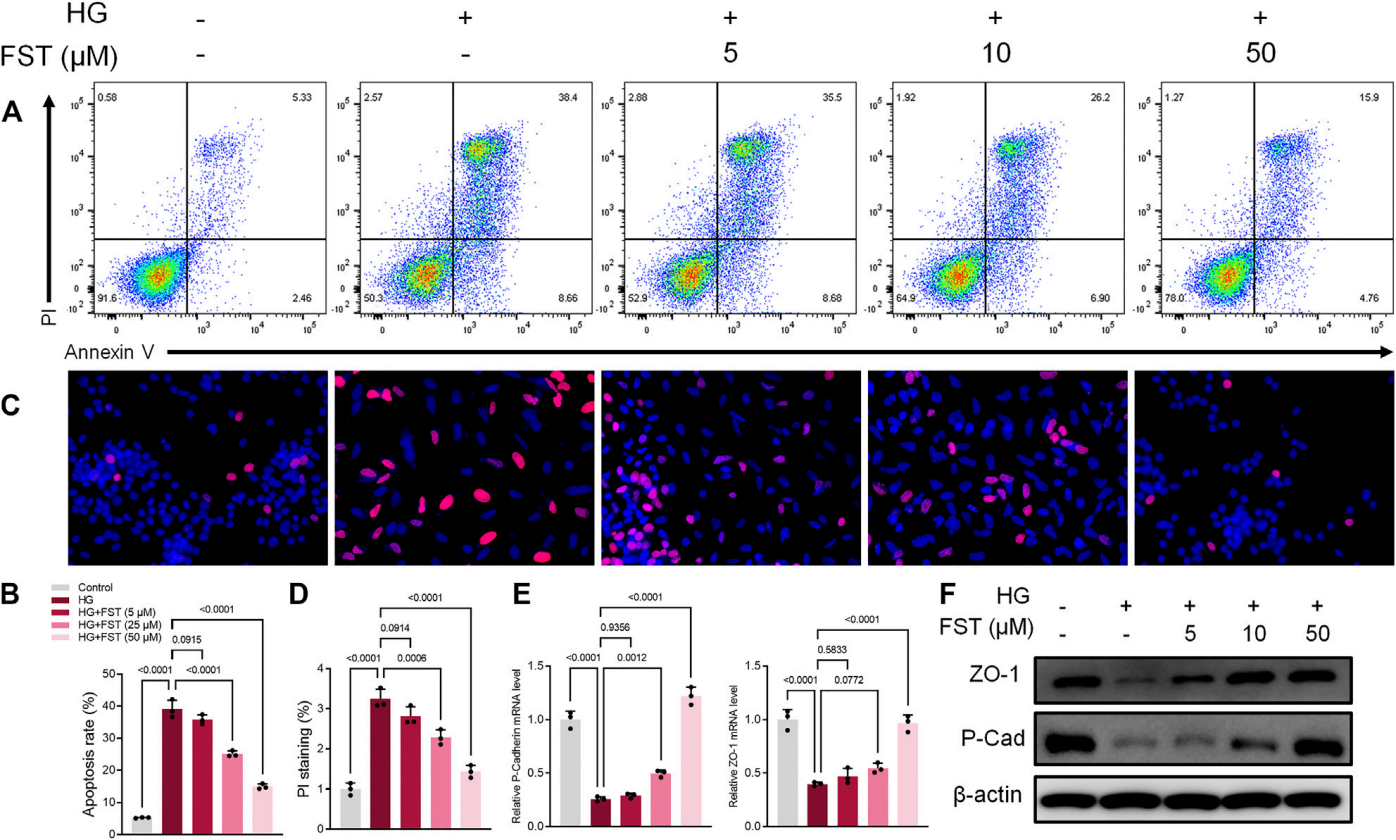
Fisetin ameliorates high glucose-induced podocyte injury. **(A,B)** Quantification and analysis of apoptosis rates using flow cytometry in podocytes after treatment with fisetin and culturing in high glucose concentrations (30 mM) for 48 h. **(C,D)** Quantification and analysis of podocytes using propidium iodide (PI) staining after treatment with fisetin and culturing in high glucose concentrations (30 mM) for 48 h. Red indicates PI staining while blue indicates DAPI staining. **(E,F)** Determination of the expression levels of P-cadherin and ZO-1 in podocytes using real-time quantitative reverse transcription polymerase chain reaction (qRT-PCR) and western blot after treatment with fisetin, and culturing in high glucose concentrations (30 mM) for 48 h. Data are expressed as means ± SEM (*n* = 3).

DN is characterized by injury to podocytes, which could be attributed to high glucose (HG) levels that are known to induce apoptosis in podocytes (Susztak et al., 2006). Therefore, we cultured the immortalized mouse podocytes in various concentrations of fisetin and high glucose concentrations (30 mM) for 48 h to determine the effect of fisetin. HG levels caused apoptosis in podocytes, while fisetin suppressed apoptosis in a dose-dependent manner based on fluorescence-activated cell sorting analysis (Figures 1A,B). In addition, cell death was assessed using propidium iodide (PI) staining. The results of the anti-apoptotic effects of fisetin are illustrated in Figures 1C,D. The occurrence of epithelial-mesenchymal transition (EMT) in podocytes can be demonstrated by negative regulation of the expression of biomarkers, such as P-cadherin and ZO-1. Therefore, we assessed the mRNA and protein expression levels of P-cadherin, ZO-1, and nephrin in the podocytes of mice. The results revealed that the expressions of P-cadherin and ZO-1 decreased considerably at mRNA and protein levels after HG treatment (Figures 1E,F), which suggests that HG accelerated the EMT process in podocytes. As expected, fisetin reversed HG-induced inhibition and attenuated HG-induced function loss in podocytes.

### Fisetin Activates CDKN1B and P70S6K-Mediated Autophagy in HG-Induced Podocyte Injury

**FIGURE 2:**
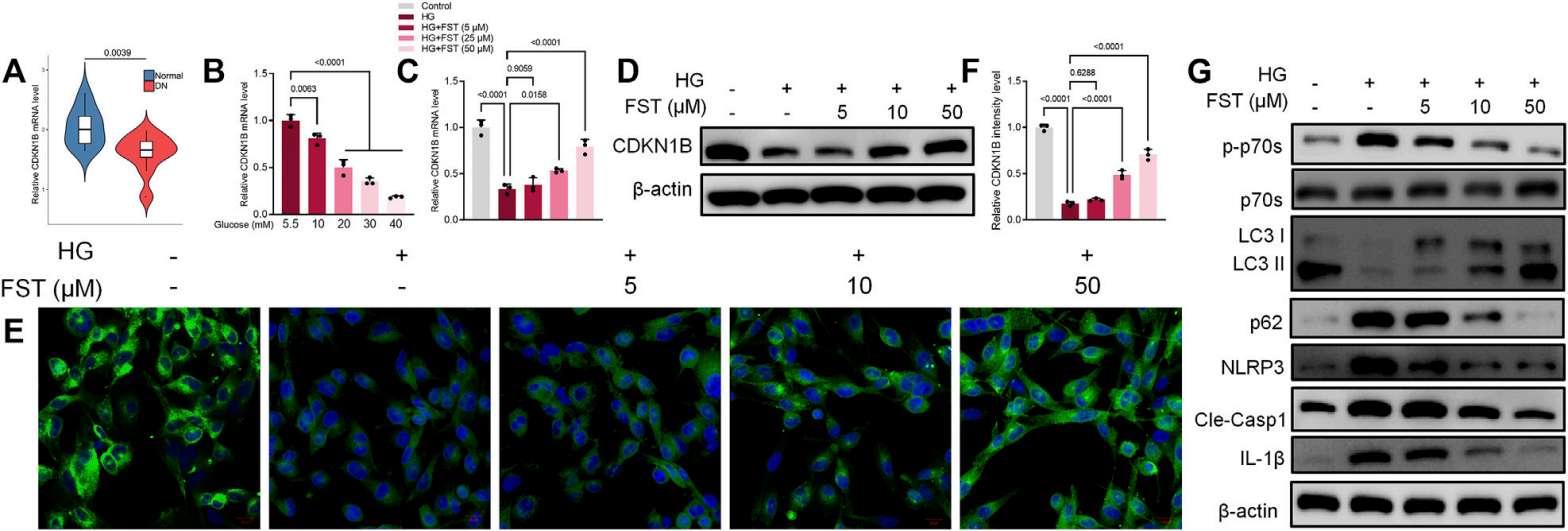
Fisetin restored protein expression levels of *CDKN1B* in podocytes cultured in high glucose concentrations. **(A)** Nephroseq dataset showing the expression levels of *CDKN1B* in normal tissues (top panels) and in diabetic kidney disease (DKD). **(B)** The expression levels of *CDKN1B* in podocytes cultured in different concentrations of glucose was determined using qRT-PCR. **(C,D)** Determination of the expression levels of *CDKN1B* in podocytes using qRT-PCR and western blot after treatment with fisetin and culturing in high glucose concentrations (30 mM) for 48 h. **(E,F)**. Immunofluorescence staining of CDKN1B in podocytes after treatment with fisetin and culturing in high glucose concentrations (30 mM) for 48 h. Green indicates CDKN1B staining and blue indicates DAPI staining. **(G)** Determination of the expression levels of phosphorylated P70S6K, P70S6K, LC3, p62, NLRP3, cleaved caspase-1, and IL-1β in podocytes using western blot after treatment with fisetin and culturing in high glucose concentrations (30 mM) for 48 h. Data are expressed as means ± SEM (*n* = 3).

The CDKN1B, also known as p27, is a potent inhibitor of the cyclin-dependent kinase that drives the G1/S transition. CDKN1B protein is thought to be a tumor suppressor with a haploid phenotype, and CDK2 activity is increased in the absence of p27 (Fero et al., 1998). However, no association of Fisetin with CDKN1B has been reported, and the mechanism of action of CDKN1B in renal disease, particularly diabetic nephropathy, is still unclear. Based on data obtained from the Nephroseq database (https://www.nephroseq.org/), *CDKN1B* mRNA expression in the glomeruli of patients with diabetic kidney disease (DKD) decreased significantly when compared with healthy individuals (Figure 2A). Furthermore, our results revealed that *CDKN1B* mRNA expression levels in podocytes decreased gradually with an increase in glucose concentrations (Figure 2B). Subsequently, we evaluated the effect of fisetin on *CDKN1B* expression. The results of reverse transcription polymerase chain reaction (RT-PCR) revealed that fisetin increased mRNA expression levels of *CDKN1B* in HG-cultured podocytes (Figure 2C). The results of western blot and immunofluorescence assays (Figures 2D–F) were consistent with RT-PCR results, which revealed that fisetin reversed the protein expression levels of *CDKN1B* in HG-cultured podocytes. Nowosad et al. (2020) reported that *CDKN1B* regulated the downstream mTOR/P70S6K pathway and autophagy. In the present study, western blot results revealed that HG levels promoted the phosphorylation of P70S6K (Figure 2G). Similarly, the results for the proteins associated with autophagy, such as light chain 3 (LC3) and P62 (SQSTM1) revealed that LC3-II/LC3-I ratio decreased, while P62 increased, suggesting autophagy deficiency. However, fisetin treatment inhibited the phosphorylation of P70S6K and promoted activation of autophagy in podocytes. Moreover, a previous study reported that activation of the Nod-like receptor protein 3 (NLRP3) inflammasome was observed in HG-induced podocytes and db/db mice. The inhibition of NLRP3 inflammasome activation is a potential target for DN treatment (Wu et al., 2021). To elucidate the role of NLRP3 inflammasome in fisetin treatment, we analyzed the levels of NLRP3, cleaved caspase-1, and IL-1β. The results revealed that fisetin reduced the protein levels of NLRP3, cleaved caspase-1, and IL-1β in a dose-dependent manner (Figure 2G), which suggests that fisetin inhibited NLRP3 inflammasome activation in podocytes.

**FIGURE 3:**
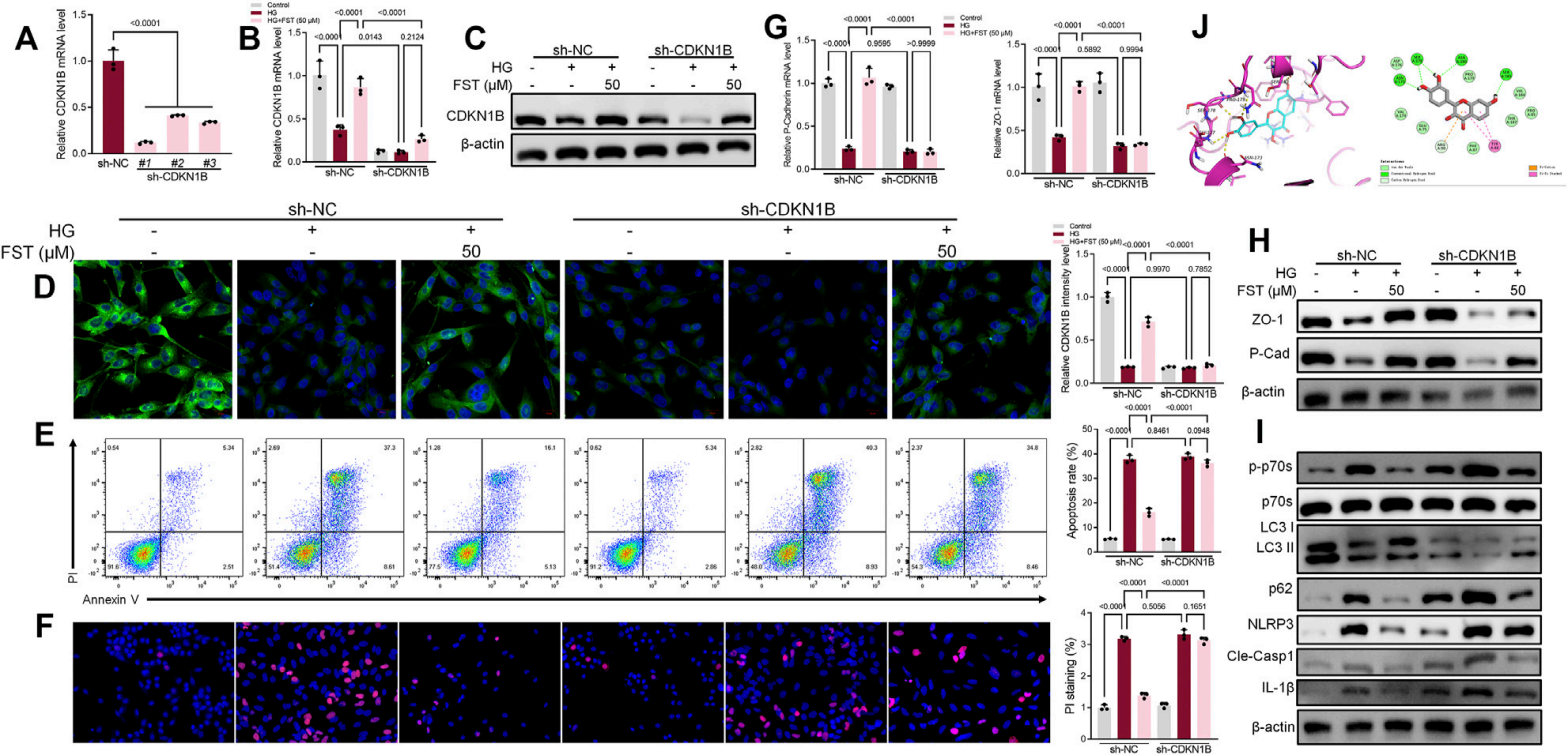
Suppression of *CDKN1B* expression counteracted the effect of fisetin on podocytes cultured in high glucose concentrations. **(A)** Determination of the expression levels of *CDKN1B* in podocytes infected with lentiviruses expressing control shRNA and three CDKN1B shRNA for 48 h using qRT-PCR. **(B,C)**. Determination of the expression levels of *CDKN1B* in podocytes transfected with CDKN1B shRNA using qRT-PCR and western blot after treatment with fisetin and culturing in high glucose concentrations (30 mM) for 48 h. **(D)** Immunofluorescence staining of CDKN1B in podocytes transfected with CDKN1B shRNA after treatment with fisetin and culturing in high glucose concentrations (30 mM) for 48 h. Green indicates CDKN1B staining while blue indicates DAPI staining. **(E)** Quantification and analysis of apoptosis rates using flow cytometry in podocytes transfected with CDKN1B shRNA after treatment with fisetin and culturing in high glucose concentrations (30 mM) for 48 h. **(F)** Quantification and analysis of podocytes transfected with CDKN1B shRNA after treatment with fisetin and culturing in high glucose concentrations (30 mM) for 48 h using propidium iodide (PI) staining. Red indicates PI staining while blue indicates DAPI staining. **(G,H)** Determination of the expression levels of P-cadherin and ZO-1 in podocytes transfected with CDKN1B shRNA using qRT-PCR and western blot after treatment with fisetin and culturing in high glucose concentrations (30 mM) for 48 h. **(I)** Determination of the expression levels of phosphorylated P70S6K, P70S6K, LC3, p62, NLRP3, cleaved caspase-1, and IL-1β in podocytes transfected with CDKN1B shRNA using western blot after treatment with fisetin and culturing in high glucose concentrations (30 mM) for 48 h. **(J)** The docking mode of FST in the binding site of CDKN1B (shown in ribbon representation and colored by structural elements). Data are expressed as means ± SEM (*n* = 3).

Podocytes were infected with lentiviruses expressing control shRNA and three CDKN1B shRNA (sh-CDKN1B) to validate the mechanisms of CDKN1B in podocytes. RT-PCR results revealed that sh-CDKN1B#1 exhibited the highest interference efficiency and was selected for subsequent experiments (Figure 3A). The mRNA expression levels of *CDKN1B* reduced further after simultaneous transfection with sh-CDKN1B and HG culture (Figure 3B). The results of western blot and immunofluorescence assays (Figures 3C,D) were consistent with those of RT-PCR, which revealed that sh-CDKN1B decreased the protein expression levels of *CDKN1B*. Furthermore, sh-CDKN1B inhibited the effect of fisetin on the improvement of HG-induced podocyte apoptosis (Figures 3E,F). Afterwards, we evaluated the maker for podocyte function. The results revealed that downregulation of *CDKN1B* suppressed the levels of P-cadherin and ZO-1, in addition to inhibiting the effect of fisetin on restoring the function of podocytes (Figures 3G,H). Moreover, western blot results revealed that the activation of P70S6K-mediated autophagy and inhibition of NLRP3 inflammasome induced by fisetin was reversed by sh-CDKN1B. Figure 3J shows the structural formula of DHK. Molecular docking studies demonstrated that FST interacted with the active site of CDKN1B with a binding energy of −6.848 kcal/mol. FST formed hydrogen bonds with the amino acid Asn173, Gly177, Ser178, Pro179 and Ser183 of CDKN1B.

**FIGURE 4:**
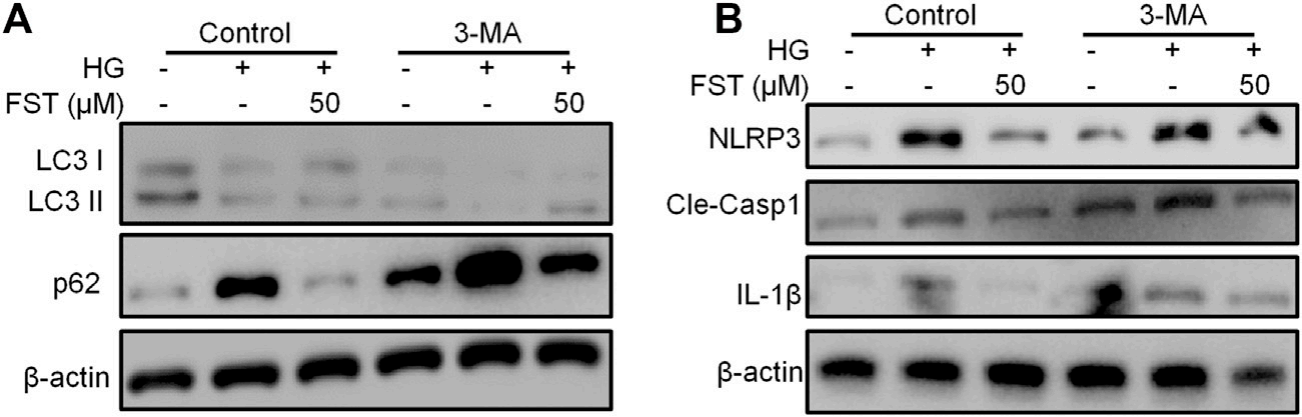
Suppression of autophagy counteracted the effect of fisetin on NLRP3 inflammasome. **(A,B)**. Determination of the expression levels of phosphorylated P70S6K, P70S6K, LC3, p62, NLRP3, cleaved caspase-1, and IL-1β in podocytes using western blot after treatment with fisetin and 3-MA (5 mM), and culturing in high glucose concentrations (30 mM) for 48 h.

Several studies have demonstrated the relationship between NLRP3 inflammasome and autophagy (Cao et al., 2019; Biasizzo and Kopitar-Jerala, 2020). Therefore, we used 3-Methyladenine (3-MA) in the present study, which inhibits autophagosome formation, to determine the relationship between NLRP3 inflammasome and autophagy in HG-cultured podocytes. The results of western blot analysis revealed that LC3-II/LC3-I ratio decreased, while P62 increased after treatment with 3-MA. The results suggest that 3-MA inhibited autophagy in podocytes (Figure 4A). Similarly, the protein levels of NLRP3, cleaved caspase-1, and IL-1β decreased (Figure 4B). The results suggest that the inhibition of NLRP3 inflammasome by fisetin is achieved through autophagy activation, and inhibition of autophagy is followed by activation of NLRP3 inflammasome.

### Fisetin Ameliorates Renal Injury in STZ-Induced Diabetic Mice

**FIGURE 5:**
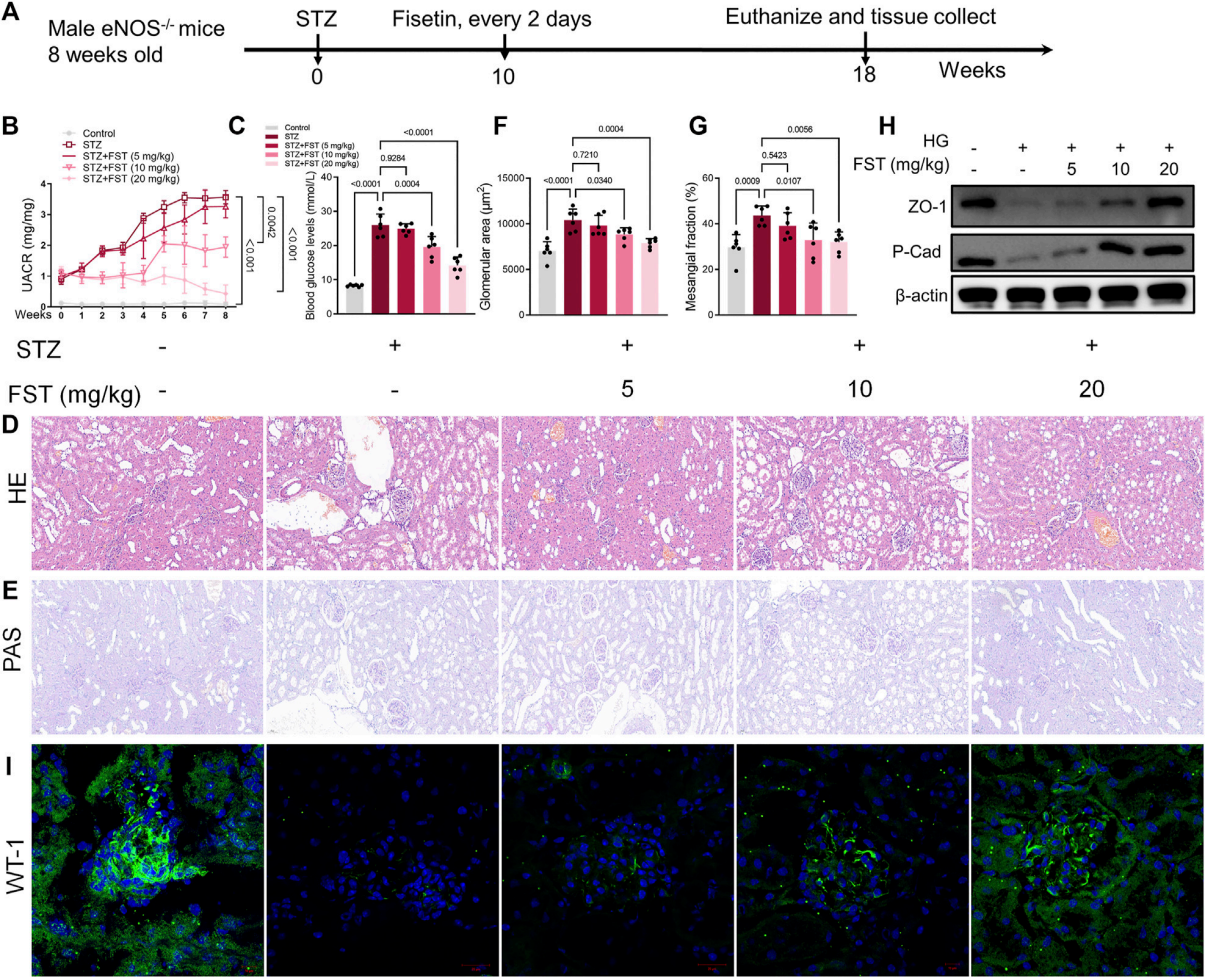
Fisetin ameliorates kidney injury in type 1 diabetic mice. **(A)** Male eNOS^−/−^ mice were injected intraperitoneally (i.p.) with 50 mg/kg STZ after 6 h of food deprivation daily for five consecutive days to induce diabetes. After 10 weeks, fisetin was orally administered to the mice every 2 days for 8 weeks and then the mice were euthanized. **(B)** Urinary albumin-to-creatinine ratios (UACR) in mice. **(C)** The level of blood glucose in mice. **(D,E)** Representative images of hematoxylin and eosin (H&E; ×200) and periodic acid–Schiff (PAS; ×200) stained sections of kidney tissues. Scale bar = 100 μm. **(F,G)** Quantification of the glomerular and mesangial matrix area fractions in mice kidney tissues. **(H)** Determination of the expression levels of P-cadherin and ZO-1 in mice kidney tissues. **(I)** Representative images of immunofluorescence-stained sections of WT-1 (×200) of mice kidney tissues. Data are expressed as means ± SEM (*n* = 6).

To elucidate the effect of fisetin in DN *in vivo*, eNOS−/− mice were injected intraperitoneally with 50 mg/kg STZ after 6 h of food deprivation daily for five consecutive days to induce diabetes. After 10 weeks, fisetin was administered orally to the mice every 2 days for 8 weeks, after which the mice were euthanized (Figure 5A). The results revealed a significant decrease in the urine albumin/creatinine ratio (UACR) and blood glucose in mice after treatment with fisetin in a dose-dependent manner (Figures 5B,C). Changes in histomorphometric parameters were analyzed using hematoxylin and eosin (H&E), periodic acid-Schiff (PAS), and Masson staining of the renal tissues in mice with DN (Figures 5D,E). The histomorphometric parameters included enlargement of the glomerular and mesangial matrix area in mice treated with STZ, an effect that was reversed by treatment with fisetin (Figures 5F,G). Thereafter, the levels of ZO-1 and P-Cadherin in renal tissues were evaluated. Fisetin treatment increased ZO-1 and P-Cadherin protein levels in the renal tissues of mice with DN (Figure 5H). Consistent with the observations, immunofluorescence assay results of Wilm’s tumor-1 (WT1) protein expression revealed that fisetin ameliorated the loss of podocytes in mice with DN (Figure 5I).

**FIGURE 6:**
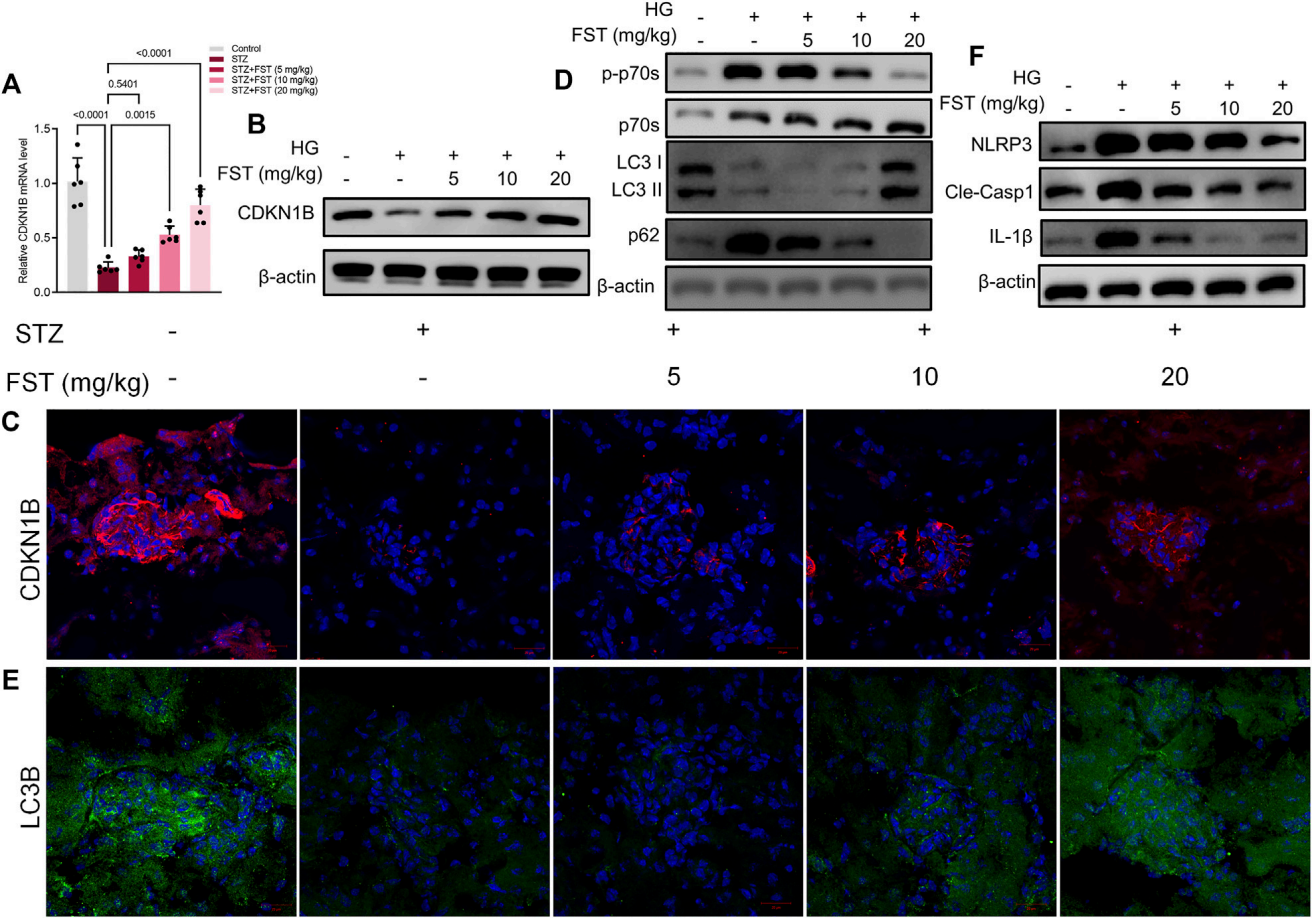
Fisetin restored protein expression levels of *CDKN1B* in type 1 diabetic mice. **(A)** Determination of the expression levels of *CDKN1B* in mice kidney tissues using qRT-PCR. **(B)** Determination of the expression levels of CDKN1B in mice kidney tissues using western blot. **(C)** Representative images of immunofluorescence-stained sections of CDKN1B (×200) of mice kidney tissues. **(D)** Determination of the expression levels of phosphorylated P70S6K, P70S6K, LC3 and p62 in mice kidney tissues using western blot. **(E)** Representative images of immunofluorescence-stained sections of LC3B (×200) of mice kidney tissues. **(F)** Determination of the expression levels of NLRP3, cleaved caspase-1, and IL-1β in mice kidney tissues using western blot. Data are expressed as means ± SEM (*n* = 6).

We analyzed the mRNA and protein expression levels of *CDKN1B* in renal tissues of mice. The results revealed that STZ reduced *CDKN1B* expression at mRNA and protein levels, while fisetin treatment stimulated *CDKN1B* expression, which was consistent with *in vitro* results (Figures 6A–C). Moreover, western blot and immunofluorescence assay results revealed that fisetin promoted P70S6K-mediated autophagy (Figures 6D,E) and blocked NLRP3 inflammasome activation in renal tissues of mice (Figure 6F). The results demonstrated that fisetin ameliorated STZ-induced DKD by stimulating autophagy and inhibiting NLRP3 inflammasome activation.

## Discussion

The pathogenesis of DN is complex and the current standard treatment for early DN is regulation of blood glucose levels and blood pressure with the renin -angiotensin-aldosterone system. However, even with such an aggressive treatment regimen, DN still progresses and eventually develops into glomerulosclerosis, renal fibrosis, and ESRD, in turn resulting in an irreversible condition (Lin et al., 2018; Ahmad et al., 2019). Therefore, there is an urgent need to develop new drugs and identify therapeutic targets for the early treatment of DN. Podocytes are highly differentiated and non-dividing cells that play a key role in maintaining the glomerular filtration barrier during the early stages of DM. Therefore, studying the mechanisms of podocyte injury in DN will provide insights into the clinical diagnosis and treatment of early DN. The present study revealed that fisetin was a natural agonist of CDKN1B that has potential renal protective effects in both type 1 diabetic animal models and HG-induced podocyte injury. To the best of our knowledge, this is the first study to elucidate detailed cellular and molecular mechanisms of fisetin in providing protection in kidney disease.

Previous studies have revealed that regulation of autophagy is a key factor influencing podocyte injury (Tagawa et al., 2016). Autophagy is the process of clearing misfolded or damaged proteins and organelles, and it is an evolutionarily conserved phenomenon from yeast to mammals (Kroemer et al., 2010). The process of autophagy begins with the formation of double-membrane vesicles, which are autophagic vesicles, formed after engulfing of misfolded proteins and damaged organelles. Autophagic vesicles bind to endophagosomes to form intermediate bisomes, which, in turn, fuse with lysosomes to form autolysosomes. The autolysosomes are rich in hydrolytic enzymes that degrade autophagic cargoes and then release them into the cytoplasm for reuse (Bhattacharya et al., 2018). The formation of autophagic vesicles is the initial stage of autophagy and it works in close association with proteins encoded by associated genes during autophagy formation. Microtubule-associated protein LC3 is also a well-recognized marker of autophagy, and LC3 consists of two main isoforms, LC3-I and LC3-II. Microtubule-associated protein 1 light chain 3 (LC3-I) is a homolog of Atg8, Atg7, and Atg3 in mammals, and its binding leads to the formation of LC3-II, which is a key step in the formation of autophagosomes (Nakatogawa, 2013). LC3-II synthesis is increased considerably during autophagy, and LC3-II is localized to the autophagic membrane vesicles and is more specific on autophagy activation (Kabeya et al., 2000). Impaired autophagy has been observed in kidney tissues from patients with DN and in established DN mouse models, where autophagy influences the maintenance of intra-lysosomal homeostasis in podocytes under diabetic conditions. Impaired autophagy is also associated with podocyte loss, leading to proteinuria in patients with DN. The findings suggest a novel strategy for the treatment of proteinuria in patients with DN. Autophagy has been demonstrated to exhibit nephroprotective effects in several animal models of aging and acute kidney injury, particularly in the glomerulus (Kume et al., 20142014). Notably, post mitotic podocytes exhibit high levels of autophagy. Previous studies have revealed that autophagy plays a crucial role in maintaining the normal function of podocytes and glomeruli. We elucidated the role of autophagy in DN by examining the expression of LC3 in the kidney tissues of each group of mice. Our experimental results revealed that the expression levels of LC3-II and Beclin-1 were reduced significantly in kidney tissues of STZ-induced mice, as well as in HG-induced podocytes, suggesting that the level of autophagy was decreased significantly in mice with DN. Our findings are consistent with those of recent studies (Kitada et al., 2017; Ma et al., 2019). However, the pathogenesis of autophagy in DN is not fully understood. A few studies have revealed that fisetin has an activating effect on autophagy (Jia et al., 2019; Yang et al., 2019; Zhang et al., 2020b). Strikingly, a few studies have also reported that fisetin inhibits autophagy (Yang et al., 2012; Sundarraj et al., 2020). We speculate that different doses of fisetin have varying effects on the regulation of autophagy in different cells. In the present study, the results showed that the autophagy marker, LC3 was significantly upregulated after fisetin activated CDKN1B and regulated the downstream target, P70S6K. The results were statistically significant in the fisetin group when compared to the model group. Therefore, it can be concluded that fisetin can regulate the level of autophagy in mice with DN and in HG-induced podocytes.

NLRP3 inflammasome was first described by Tschopp et al., in 2002 and is thought to be essential in natural immunity (Martinon et al., 2002). As a key member of the Nod-like receptor (NLR) family, inflammasomes are closely associated with the development and progression of DN. The expression levels of NLRP3, caspase-1, apoptosis-associated speck-like protein, and IL-1 is-associated in patients with DN are considerably high, resulting in an inflammatory response in the body, which, in turn, leads to excessive production of ECM and various types of cell necrosis, and programmed cell death (Jo et al., 2016). NLRP3 inflammasome recognizes pathogen-associated molecular patterns (PAMPs) and danger-associated molecular patterns (DAMPs). Afterwards, NLRP3 activates a series of downstream signaling pathways, which leads to the cleavage of inactive IL-1β and IL-18 precursors into mature and active IL-1β and IL-18, which are subsequently released into the extracellular compartment, causing inflammatory responses and oxidative stress in the body. Overexpression of IL-1β and IL-18 can have an impact on the kidneys, resulting in a series of pathological changes in the kidney, and clinical manifestations associated with renal impairment. Serum creatinine, blood urea nitrogen, and UACR in patients with DN were significantly higher in NLRP3-positive patients than in those with inhibited NLRP3 expression. The observations may be regarded as the clinical manifestations of renal impairment due to overexpression of IL-1β and IL-18 following NLRP3 inflammasome activation (Fan et al., 2019). Numerous studies have revealed that NLRP3 is a key factor influencing the development and progression of DN, and that NLRP3 inflammasome can regulate the development and progression of DN through a variety of related signaling pathways. Chronic hyperglycemia induces the expression and activation of NLRP3 inflammasome and Pro-caspase-1 in thylakoid cells, releasing IL-1β and IL-18, which influence inflammatory responses in the kidneys, and the increase in renal collagen fibrils (Feng et al., 2016). Hyperglycemia also induces the expression of *TXNIP* (thioredoxin-interacting protein), which activates NADPH oxidases to produce reactive oxygen species. Subsequently, TXNIP triggers activation of the NLRP3 inflammasome in podocytes, which, in turn, results in podocyte loss and albuminuria (Fakhruddin et al., 2017). Moreover, activation of the NLRP3 inflammasome induces loss of podocyte proteins, nephrin and podocin, as well as mitochondrial dysfunction in mice podocytes (Zhao et al., 2018). The role of NLRP3 inflammasome activation in tubular injury has been associated with an increase in HG-induced EMT and the role of SMAD3, p38 MAPK, ERK1, and ERK2 signaling pathways, which influence the pro-inflammatory and fibrotic responses in tubular cells. Phosphorylation of key signaling molecules, such as ERK and p38 MAPK can upregulate the expression of IL-18 and IL-1β. The overexpression of IL-18 and IL-1β facilitates fibrotic processes in the kidneys by promoting TGF-β activation in renal tubular epithelial cells, and ultimately resulting in the development and progression of DN (Tang and Yiu, 2020). The mechanism through which NLRP3 inflammasome causes DN is exceptionally complex, and LncRNAGm4419 has been implicated in glomerular thylakoid inflammation and fibrosis in hyperglycemic states via the NF-κB/NLRP3 inflammatory pathway, which ultimately results in the development of DN (Li et al., 2020). In addition, previous studies have revealed that spleen tyrosine kinase (Syk) is involved in hyperglycemia-induced activation of the Syk/JNK/NLRP3 signaling pathway in rat glomerular thylakoid cells and HK2 cells. Activation of the Syk/JNK/NLRP3 signaling pathway causes glomerular hypertrophy and thylakoid expansion in rats, and Syk induces apoptosis in HK2 cells, further aggravating renal injury in hyperglycemic conditions (Qiu and Tang, 2016). Our results revealed that inhibition of autophagy after HG induction in podocytes activated the NLRP3 inflammasome, which was further activated following treatment with autophagy inhibitor, 3-MA, suggesting that autophagy negatively regulates NLRP3 inflammasome activation in podocytes. In addition, previous studies have reported that fisetin can inhibit NLRP3 inflammasome through activation of autophagy (Zhang et al., 2020b). Pu et al. (2021) found that Fisetin showed protective effects against ischemia-reperfusion injury through mediating the NLRP3 inflammasome pathway 34024736. Besides, former study confirmed that Fisetin reduced periodontitis by inhibiting inflammatory reaction *via* NLRP3 (Huang et al., 2021). What’s more, Fisetin has been found to reserve the TLR4/MD2-mediated activation of NLRP3 inflammasome in a p62-dependent manner (Huang et al., 2021) which is consistent with the findings of the present study.

In conclusion, the present study revealed that fisetin alleviated HG-induced podocyte injury and STZ-induced DN in mice by restoring CDKN1B/P70S6K-mediated autophagy and inhibiting NLRP3 inflammasome. The present study provides novel insights into the reno-protective effects of fisetin in patients with DN.

## Data Availability

The raw data supporting the conclusion of this article will be made available by the authors, without undue reservation.
