# Supplementary material for: Fisetin Attenuates Diabetic Nephropathy-Induced Podocyte Injury by Inhibiting NLRP3 Inflammasome
Source: Front Pharmacol. 2022 Jan 21;13:783706. doi: 10.3389/fphar.2022.783706 (PMC8816314; doi:10.3389/fphar.2022.783706)
Supplement: Supplementary file 1 [file DataSheet1.DOCX]

**Fisetin attenuates diabetic nephropathy-induced podocyte injury by inhibiting NLRP3 inflammasome**

Wenmin Dong^1,2#^, Chenglin Jia ^1#^, Ji Li^1^, Jibo Liu^1^, Zhiguo Zhao^1^, Jiaqi Zhang ^1,2*^, Shan Lin^1*^, Ying Chen^1,2*^

*1**. Shanghai TCM-Integrated Hospital affiliated to Shanghai University of Traditional Chinese Medicine, Shanghai, 200082, China*

*2. Shanghai University of Traditional Chinese Medicine, Shanghai,201203, China*

^#^ These authors contribute equally to this work

*Correspondence：

Ying Chen

Email: lcys7382@126.com, Tel:13524922483

Shanghai TCM-Integrated Hospital affiliated to Shanghai University of Traditional Chinese Medicine, Shanghai, 200082, China

Shanghai University of Traditional Chinese Medicine, Shanghai,201203, China

Shan Lin

Email: chrislinshan@126.com Tel：13774456815

Shanghai TCM-Integrated Hospital affiliated to Shanghai University of Traditional Chinese Medicine, Shanghai, 200082, China

Jiaqi Zhang

Email: zhangjiaqinelly@gmail.com, Tel:13816549791

Shanghai TCM-Integrated Hospital affiliated to Shanghai University of Traditional Chinese Medicine, Shanghai, 200082, China

Shanghai University of Traditional Chinese Medicine, Shanghai,201203, China

**Methods**

**Cell Transfection**

For CDKN1B knockdown, sh- CDKN1B vector were constructed by GenePharma (Shanghai, China), and podocytes were transfected with sh-Sirt1 at a final concentration 50 nM using Lipofectamine 2000 (Invitrogen, Carlsbad, CA, United States) according to the manufacturer’s protocol.

**Flow Cytometry Assay**

Logarithmically growing podocytes were seeded into culture flasks. The cells were dual stained with Annexin V-FITC and propidium iodide (PI) for 30 min at room temperature. The stained cells were immediately analyzed by flow cytometry (Becton Dickinson, Franklin Lakes, NJ, United States). Apoptotic cells were defined as Annexin V-FITC positive and PI negative.

**RNA Isolation and Quantitative Real-Time PCR**

RNA was isolated using the TRIzol reagent (Invitrogen) according to the manufacturer’s instructions in podocytes and mouse kidney samples and reverse transcribed using a miScript Reverse Transcription kit (Qiagen). QRT-PCR was performed using the SYBR Premium Ex Taq II kit (Takara, Dalian, China) in an ABI PRISM 7500 Sequence Detection System (Applied Biosystems). All reactions were performed in triplicate and the mean value was used to calculate expression levels after normalization to β-actin as an internal standard.

**Protein Extraction and Western Blot Analysis**

Podocytes were lysed using RIPA buffer, and protein concentration was determined using the BCA protein assay kit. Approximately 30 μg of protein from each sample was separated using a 10% SDS-polyacrylamide gel and transferred to PVDF membranes. Membranes were blocked with 5% skim milk in TBST and incubated with primary antibodies overnight at 4°C. Membranes were then incubated with the corresponding secondary antibodies for 1 h at room temperature and washed in TBST. Proteins were detected using specific antibodies: CDKN1B (#3686), ZO-1 (#13663), P-Cadherin (#14029), Phospho-p70S6 Kinase (Thr389) (#97596), p70S6 Kinase (#9202), NLRP3 (#15101), Cleaved Caspase-1 (#89332), IL-1β (#12242), LC3B (#3868), p62 (#39749), β-Actin (#3700).

**Kidney histology**

Kidneys were removed and fixed with 4% paraformaldehyde 16 h at 4 °C. The 4-μm sections were cut from paraffin-embedded kidney tissues. Sections were stained with PAS for histology analysis. Assessment of the mesangial and glomerular cross-sectional areas was performed by pixel counts on the kidney section in a blinded fashion, under × 400 magnification (Olympus, Tokyo, Japan) as previously described32,33. In brief, digitized images were scanned and profile areas were traced using ImageJ. The mean glomerular tuft volume was determined from the mean glomerular cross-sectional area by light microscopy. The glomerular cross-sectional area was calculated based on the average area of 30 glomeruli in each group as previously described [[1](#_ENREF_1)].

**Molecular Docking Analysis**

Molecular docking analyses were performed to investigate interactions between α-Hederin and EGR1 using AutoDock Vina 1.1.2 [[2](#_ENREF_3)]. A 3D structure of α-Hederin was drawn using ChemBioDraw Ultra 14.0 and converted to a 3D structure by ChemBio 3D Ultra 14.0. The 3D coordinates of EGR1 (PDB ID: 4X9J) were retrieved from the RCSB Protein Data Bank. The homology model was obtained from SWISS-MODEL (<https://www.swissmodel.expasy.org/>). AutoDockTools version 1.5.6 [[2](#_ENREF_3), [3](#_ENREF_4)] was employed to generate docking input files. The crystallographic ligands were extracted and fed into a docking database for redocking, and hydrogen atoms added. An auxiliary program AutoGrid was used to generate a docking area that was defined as a 40 × 40 × 40 3D grid centered on the ligand binding site with a 0.375 Å grid space. All bond rotations for the ligands were ignored in this study. The best scoring pose from Vina docking score evaluations was selected for further analyses using PyMoL 1.7.6 software.

**Reference**

[1] Zhong, Y.; Lee, K.; Deng, Y.; Ma, Y.; Chen, Y., et al. Arctigenin attenuates diabetic kidney disease through the activation of PP2A in podocytes, Nature communications.10 (2019) 4523, https://doi.org/10.1038/s41467-019-12433-w.

[2] Trott O, Olson AJ. AutoDock Vina: improving the speed and accuracy of docking with a new scoring function, efficient optimization, and multithreading. Journal of computational chemistry. 2010;31:455-61.

[3] Sanner MF. Python: a programming language for software integration and development. Journal of molecular graphics & modelling. 1999;17:57-61.
